# Supplementary material for: Ameliorative Effect of Structurally Divergent Oleanane Triterpenoid, 3-Epifriedelinol from Ipomoea batatas against BPA-Induced Gonadotoxicity by Targeting PARP and NF-κB Signaling in Rats
Source: Molecules. 2022 Dec 29;28(1):290. doi: 10.3390/molecules28010290 (PMC9822353; doi:10.3390/molecules28010290)

Supplementary Materials

S1. Isolation scheme of EFD

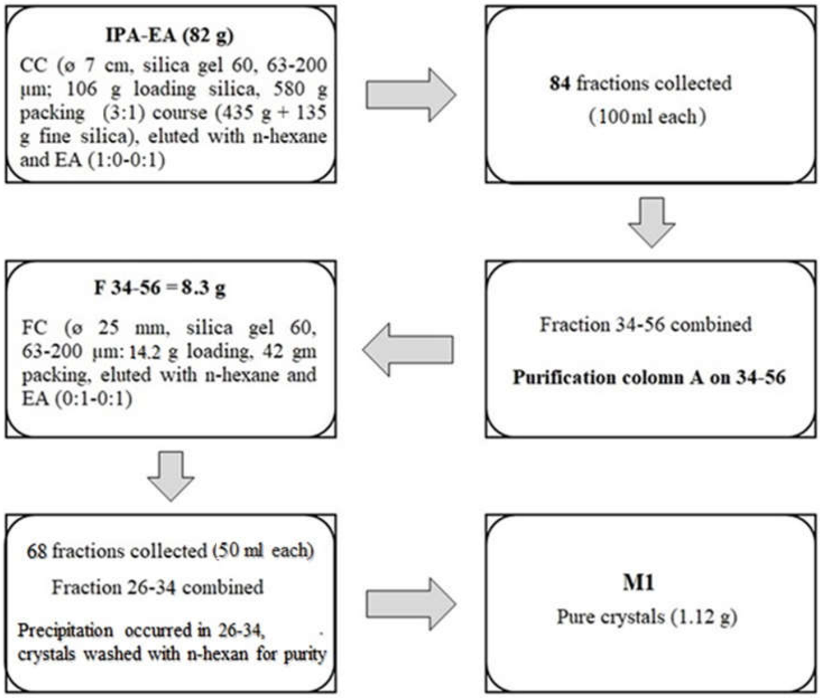

Figure S1 Schematic representation of isolation of EFD from IPA-EA.

Note: DCM: dichloromethane; EA: ethyl acetate; CC: column chromatography; FC: Flash column chromatography.

S2. The 1H and 13C spectra, XRD proposed 3D structural model

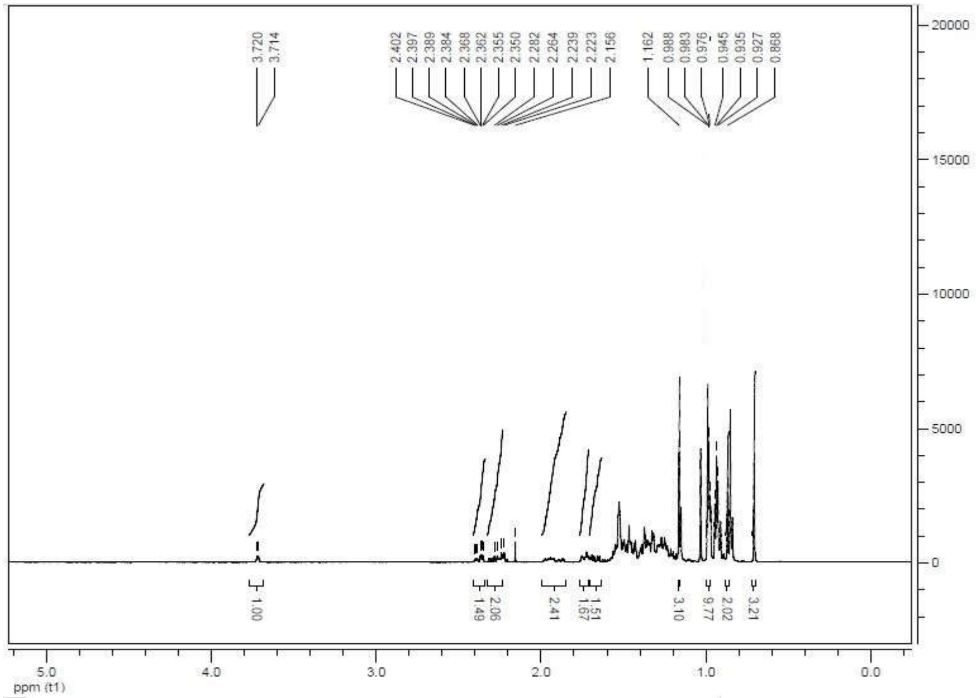

Figure S2 ^{1}H spectrum (400 MHz, in $CDCl_3$) of EFD.

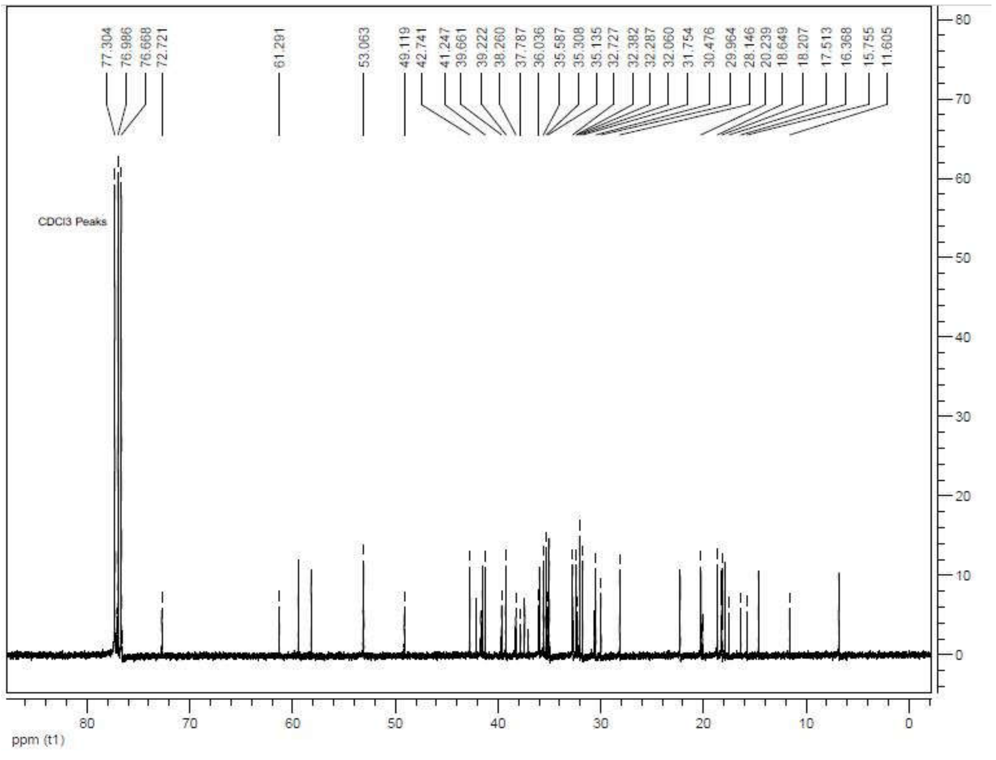

Figure S2 ^{13}C (300 MHz, in $CDCl_3$) spectrum of EFD.

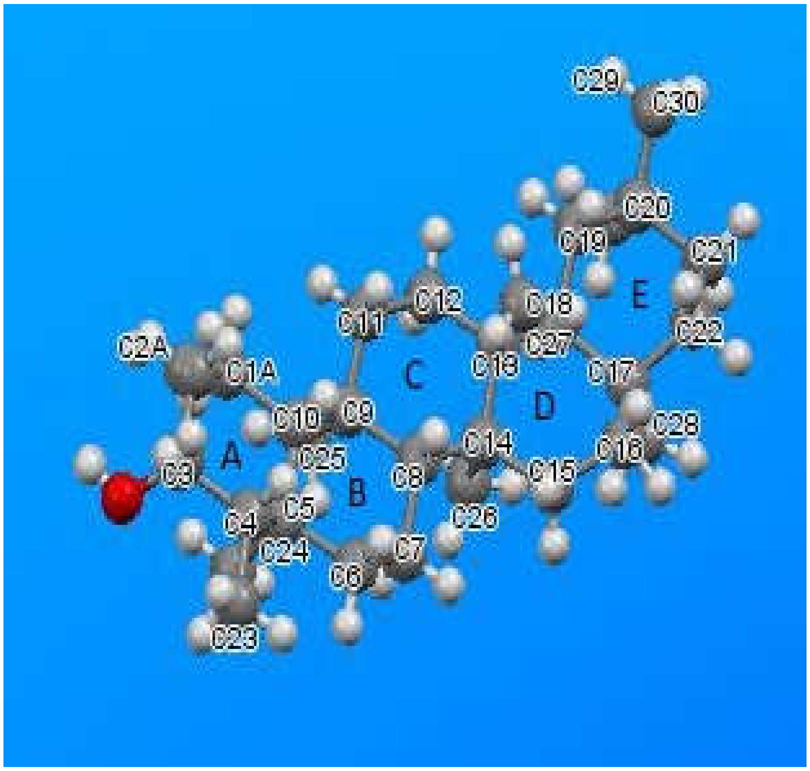

Figure S3 3D structural model of EFD as proposed by XRD.

S3. Details of ligands molecular docking and structure analysis of the target proteins

S3.1 Ligands-molecular docking

The compounds were designed in Discovery Studio Client and saved in PDB format as ligands after energy minimization. Autodock Tools was used for the preparation of ligands in their most stable conformation. After addition of the Kolman and Gasteiger charges the ligands were saved in PDBqt format. Molecular docking analysis was used for all the synthesized ligands against BAX, BCL-2, NF-κB and P53 by PyRx virtual screening tool with Auto Dock VINA Wizard approach. The grid box center values for BAX (PDBID: 2K7W) (center X = 0.273 center Y = 4.614 center Z = 2.980) and size values were adjusted (X = 126, Y = 64, Z = 62). BCL2 (center X = 226.442 center Y = 12.598 center Z = 112.161) and size values were adjusted (X = 64, Y = 108, Z = 82). NF-kB (center X = -10.754 center Y = 12.598 center Z = 112.161) and size values were adjusted (X = 116, Y = 126, Z = 116). P53 (center X = 6.586 center Y = 22.334 center Z = -0.939) for better conformational position in the active region of the target protein. Ligands were docked individually against nitric oxide synthase and angiotensin converting enzyme with default exhaustiveness value of 25. The predicted docked complexes were evaluated based on the lowest binding energy values (Kcal/mol). The 3D graphical depictions of all

the docked complexes were accomplished by Discovery Studio (2.1.0) (Discovery Studio Visualizer Software, Version 4.0., 2012).

S3.2 Structural analysis of target proteins

BAX consisted of 70% helices (148 residues), 0% β-sheets, 30% coils (63 residues), 14% turns (30 residues) and a total of 212 amino acid residues and R= 0.210. and resolution A°=1.22 . Unit cell dimensions for the lengths were observed to be: a= 69.786, b= 91.573 and c= 156.096 with 90º angle for α, β and γ. Ramachandran plot confirmed that 96 % amino acids were in the allowed regions for the phi (φ) and psi (ψ) angles. BCL2 consisted of 69% helices (101 residues), 0% β-sheets, 30% coils (45 residues), 35% turns (52 residues) and a total of 158 amino acid residues R= 0.150 and resolution A°=1.19. Unit cell dimensions for the lengths were observed to be: a= 69.786, b= 91.573 and c= 156.096 with 90º angle for α, β and γ. Ramachandran plot confirmed that 96 % amino acids were in the allowed regions for the phi (φ) and psi (ψ) angles. Nf-kB consisted of 90% helices (59 residues), 45% β-sheets (287 residues), 44% coils (278 residues), 7% turns (44 residues) and a total of 672 amino acid residues R= 0.340 and resolution A°=2.30. Unit cell dimensions for the lengths were observed to be: a= 84.2, b= 132.1 and c= 80.1 with 90º angle for α=90, β=93.1 and γ=90. Ramachandran plot confirmed that 97 % amino acids were in the allowed regions for the phi (φ) and psi (ψ) angles. P53 consisted of 64% helices (20 residues), 0% β-sheets, 35% coils (11 residues), 0% turns and a total of 31 amino acid residues R= 0.252 and resolution A°=1.50. Unit cell dimensions for the lengths were observed to be: a= 69.786, b= 91.573 and c= 156.096 with 90º angle for α, β and γ. Ramachandran plot confirmed that 93 % amino acids were in the allowed regions for the phi (φ) and psi (ψ) angles.

S3.3 2D and 3D images representing docked ligand (EFD) with target proteins

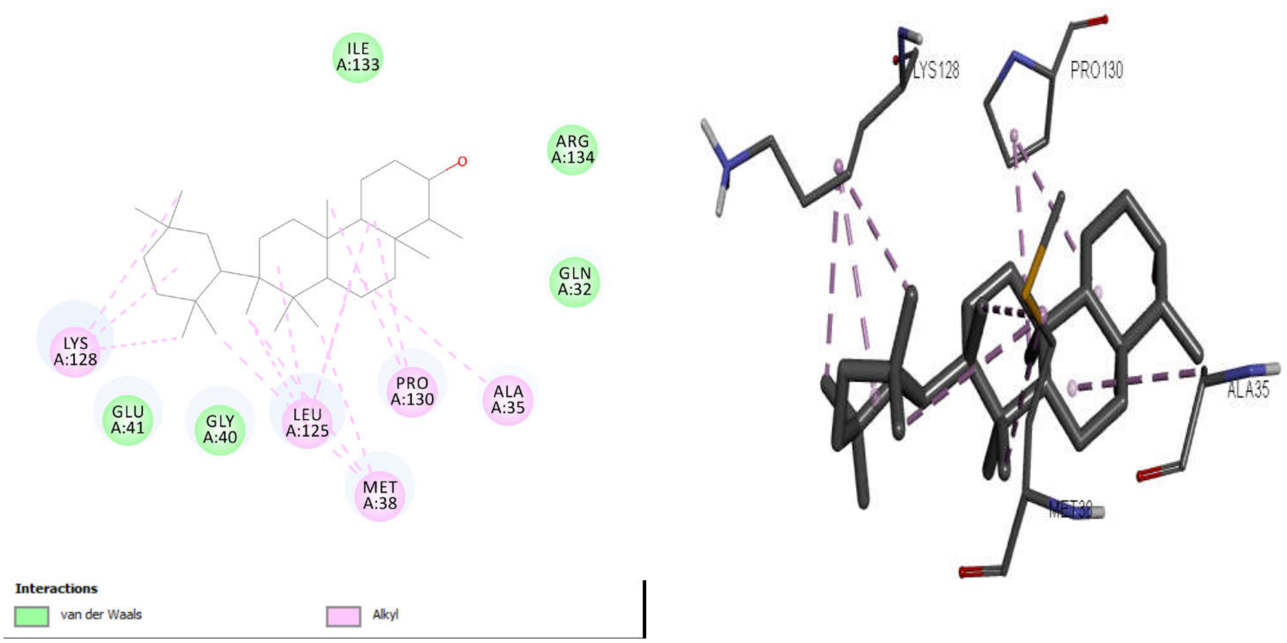

Figure S3. Representation of docked ligand with BAX (PDB-ID: 2K7W)

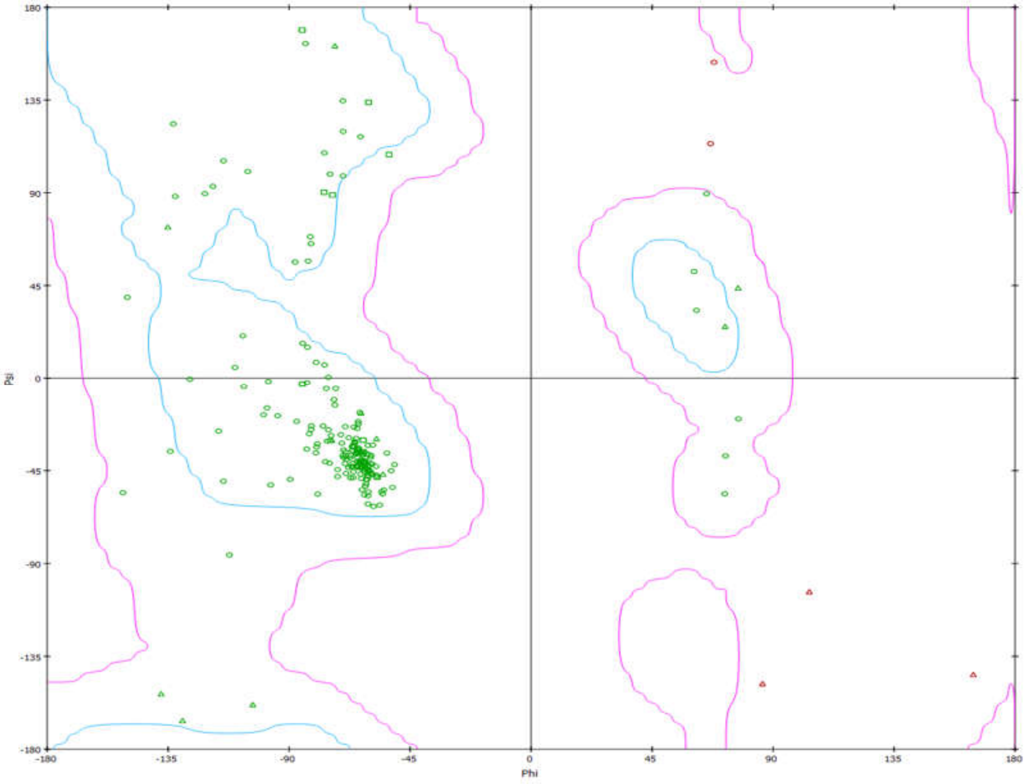

Figure S4 Ramachandran plot confirming that 96 % amino acids are in the allowed regions for the phi (φ) and psi (ψ) angles.

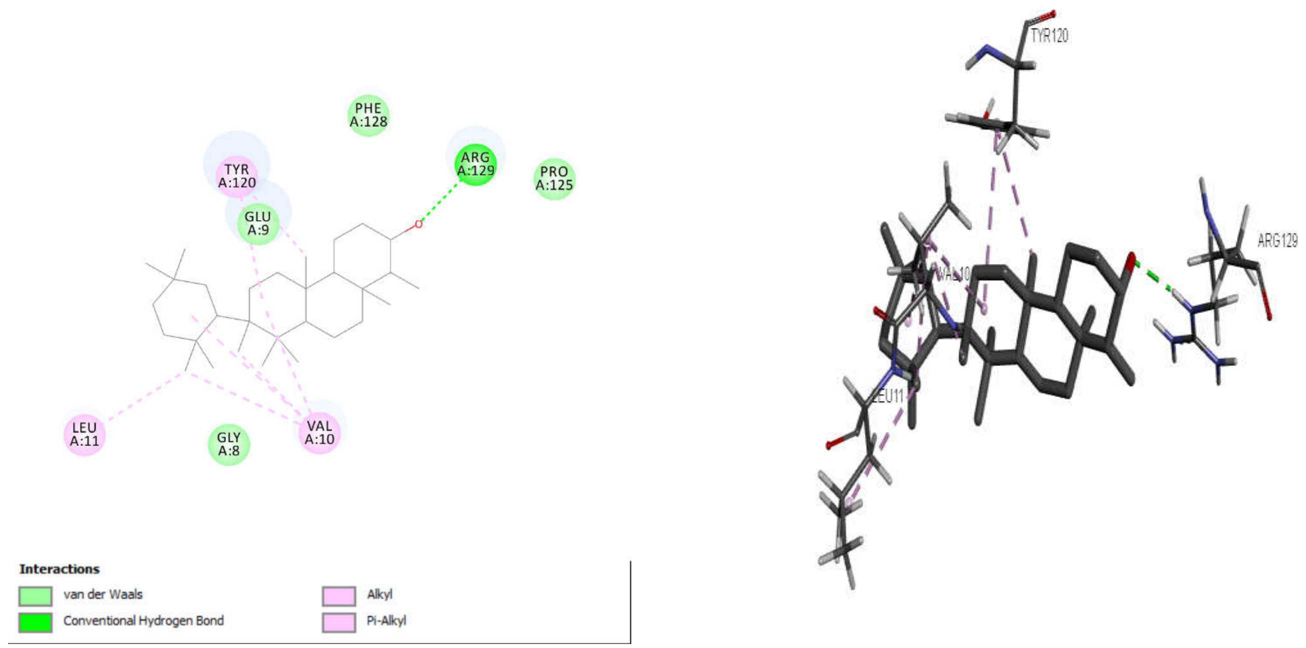

Figure S5. Representation of docked ligand with BCL-2 (PDB-ID 1K3K)

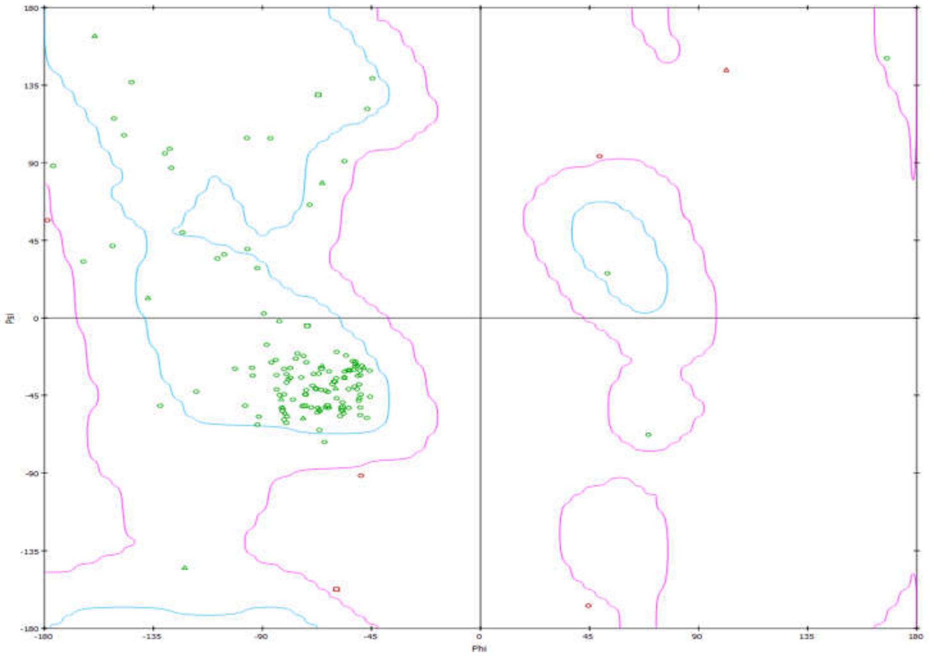

Figure S6 Ramachandran plot confirming that 96 % amino acids are in the allowed regions for the phi (φ) and psi (ψ) angles.

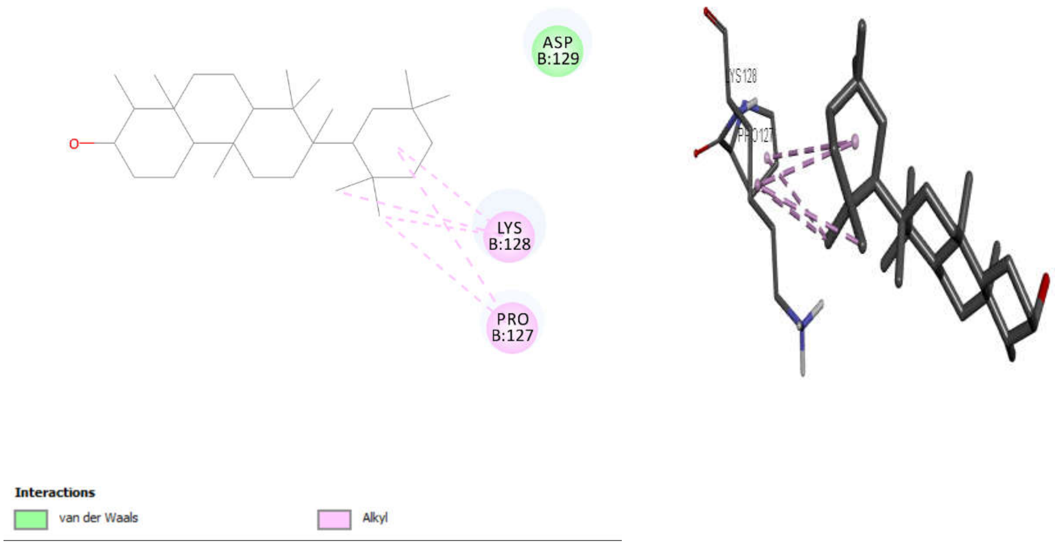

Figure S7. Representation of docked ligand with NF-kB (PDB-ID 1NFK)

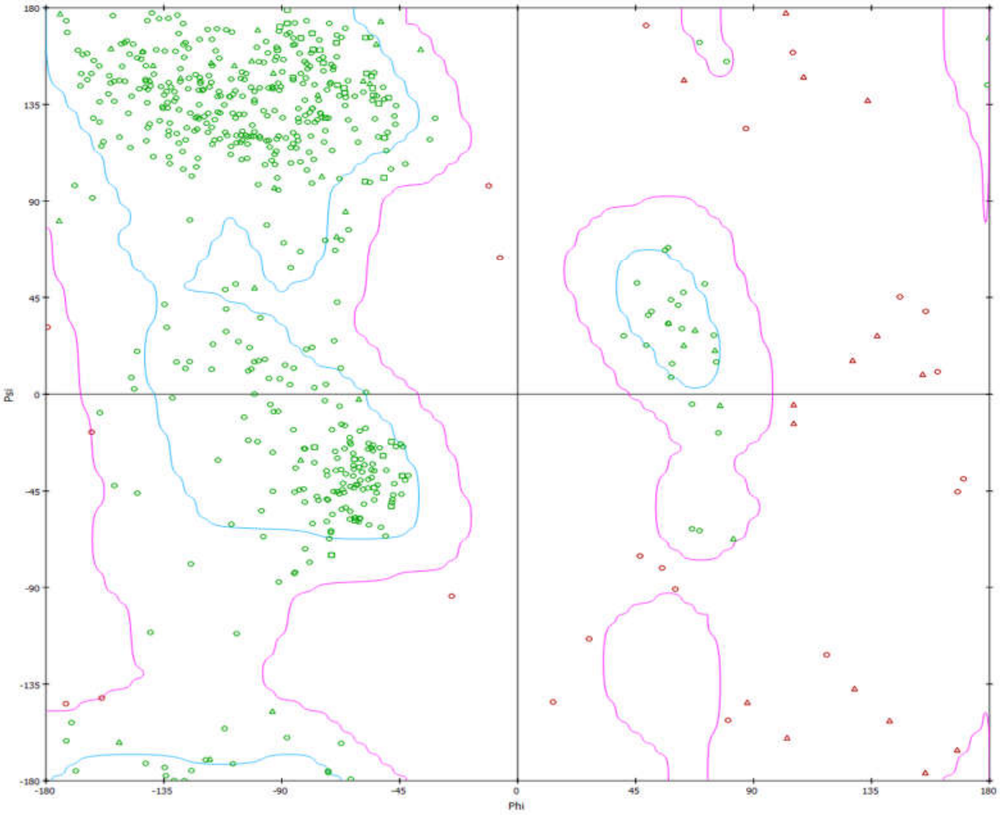

Figure S8 Ramachandran plot confirming that 96 % amino acids are in the allowed regions for the phi (φ) and psi (ψ) angles.

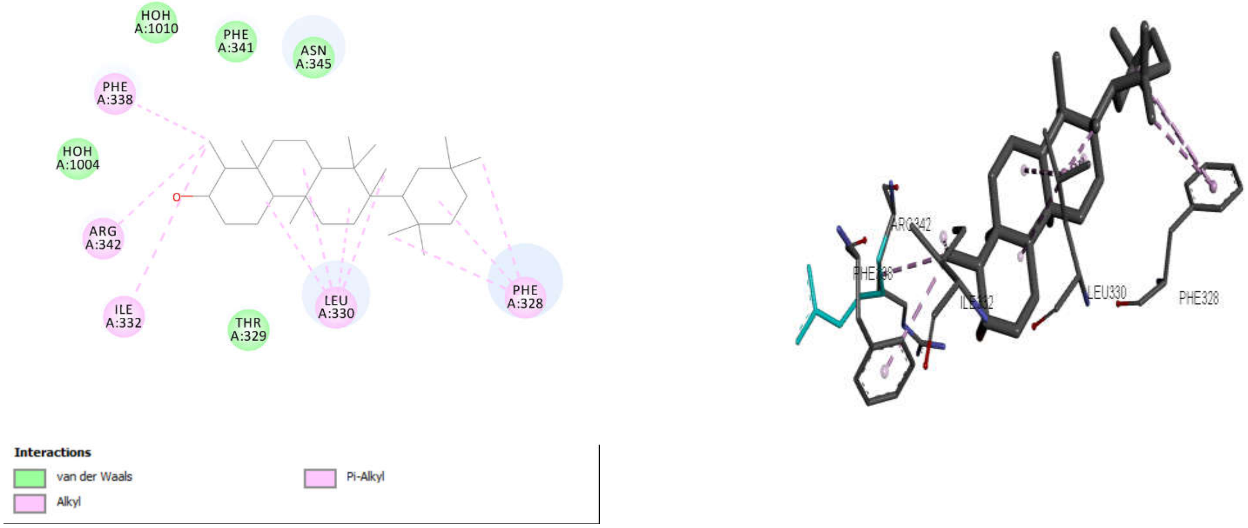

Figure S9. Representation of docked ligand with P53 (PDB-ID 1AIE)

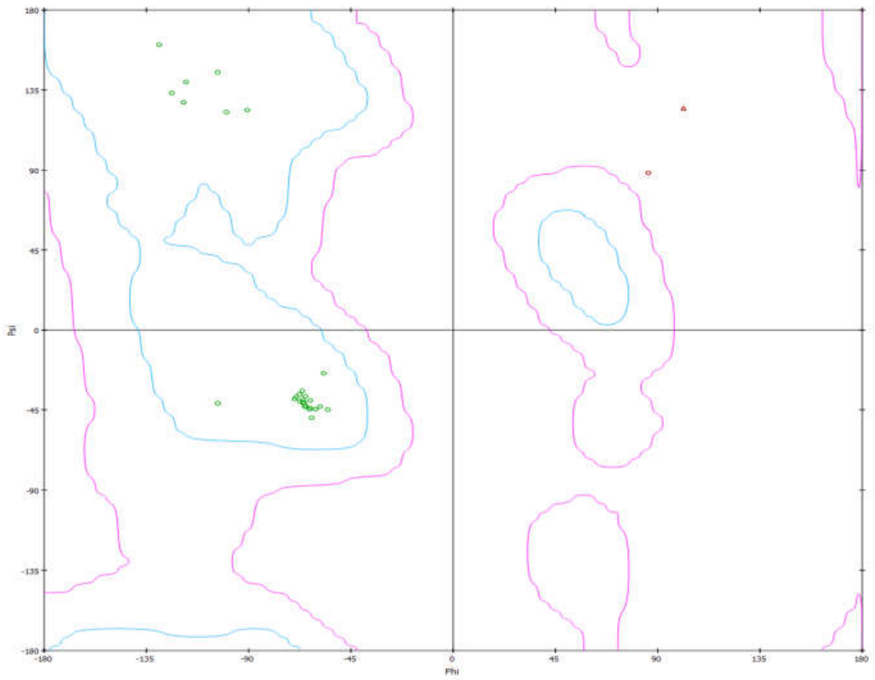

Figure S10 Ramachandran plot confirming that 96 % amino acids are in the allowed regions for the phi (φ) and psi (ψ) angles.

3.4 2D and 3D images representing docked ligand (Doxorubicin) with target proteins

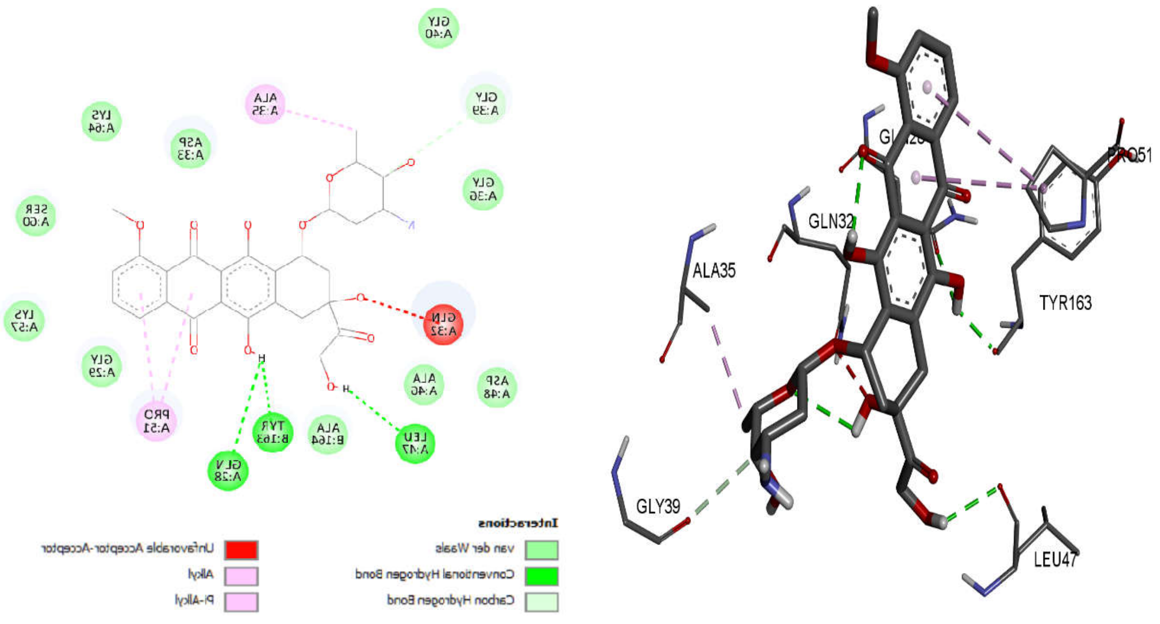

Figure S11. Representation of docked ligand with BAX (PDB-ID: 2K7W)

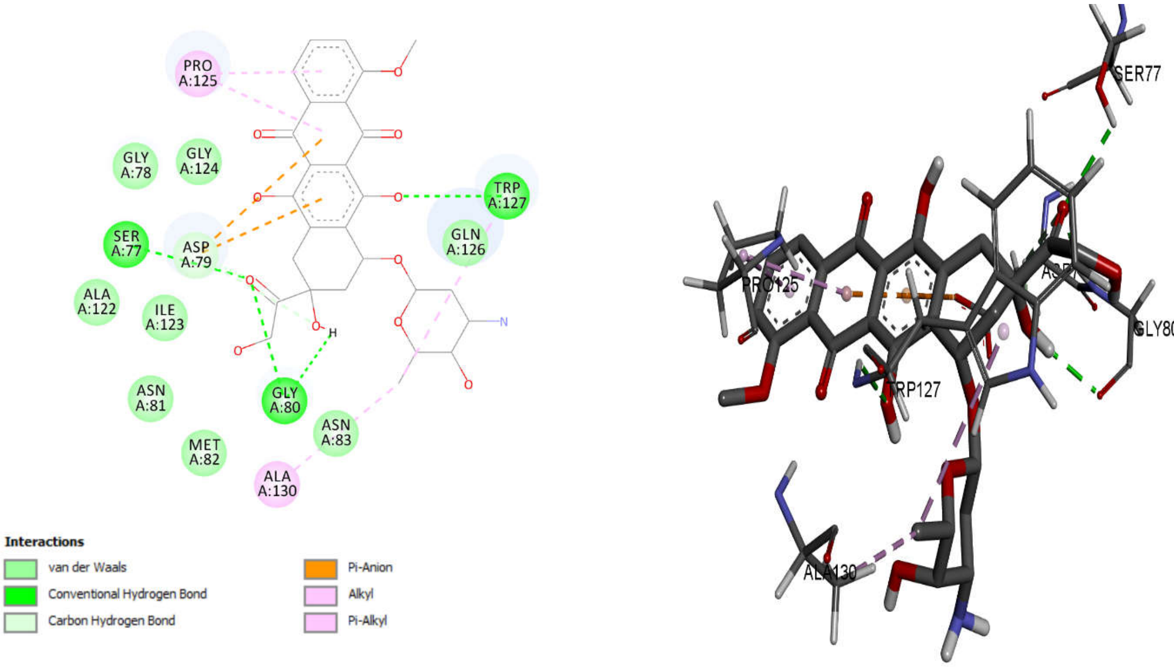

Figure S12. Representation of docked ligand with BCL-2 (PDB-ID 1K3K)

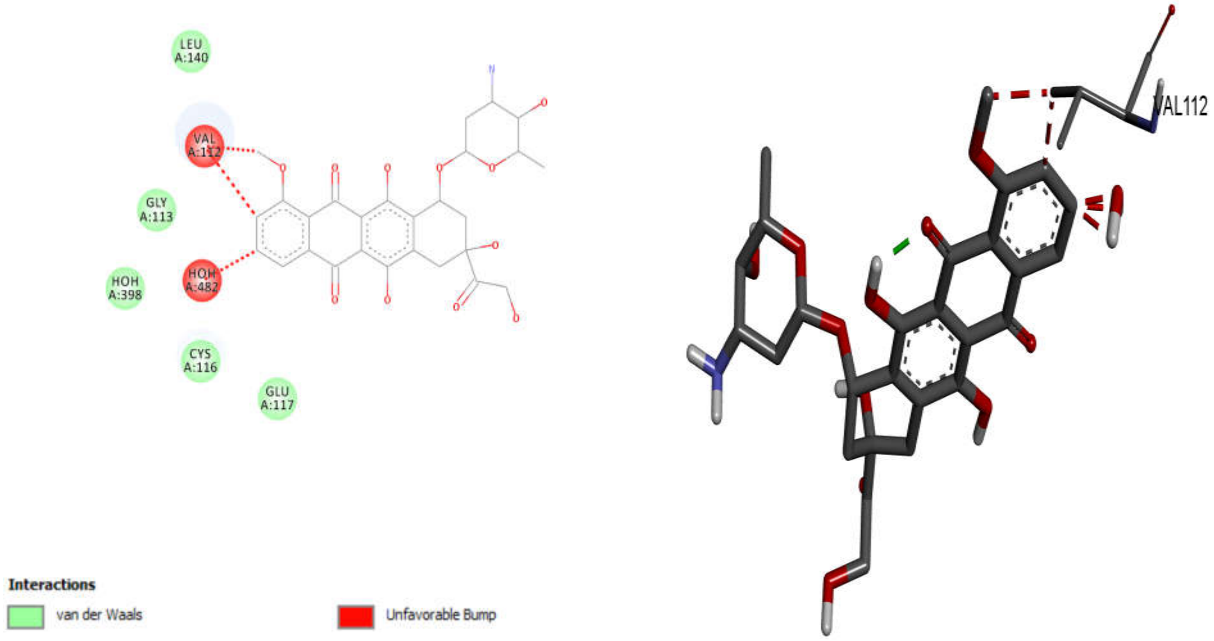

Figure S13. Representation of docked ligand with NF-kB (PDB-ID 1NFK)

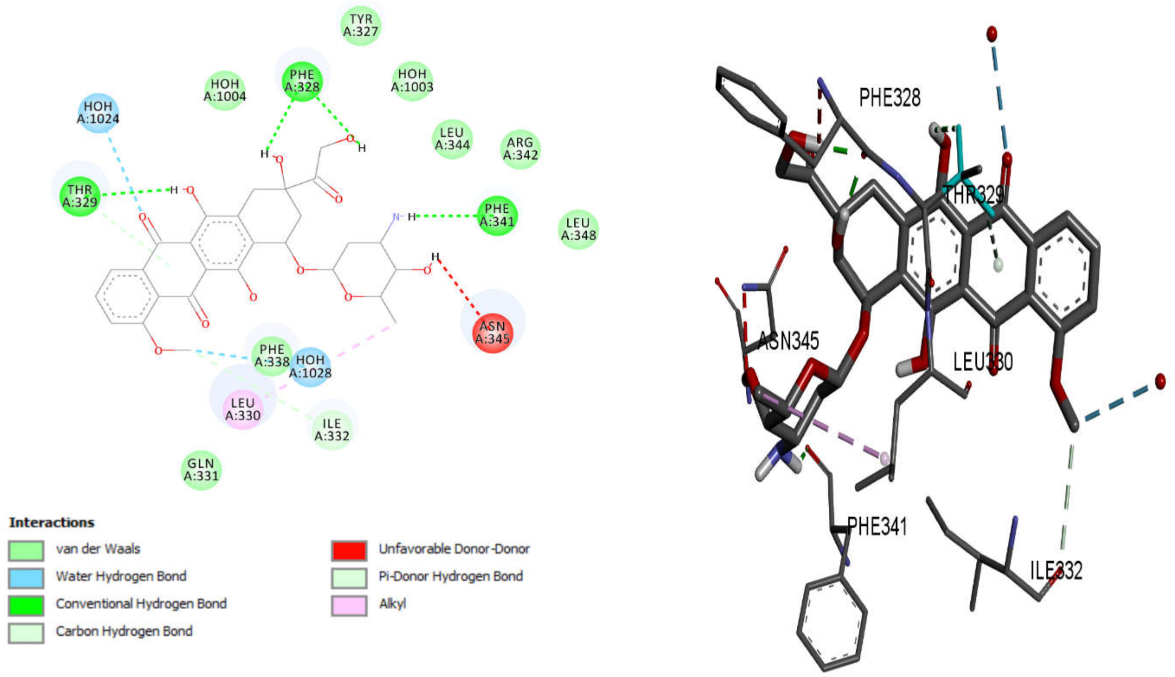

Figure S14. Representation of docked ligand with P53 (PDB-ID 1AIE)

4. Survival rate with time curve after acute toxicity

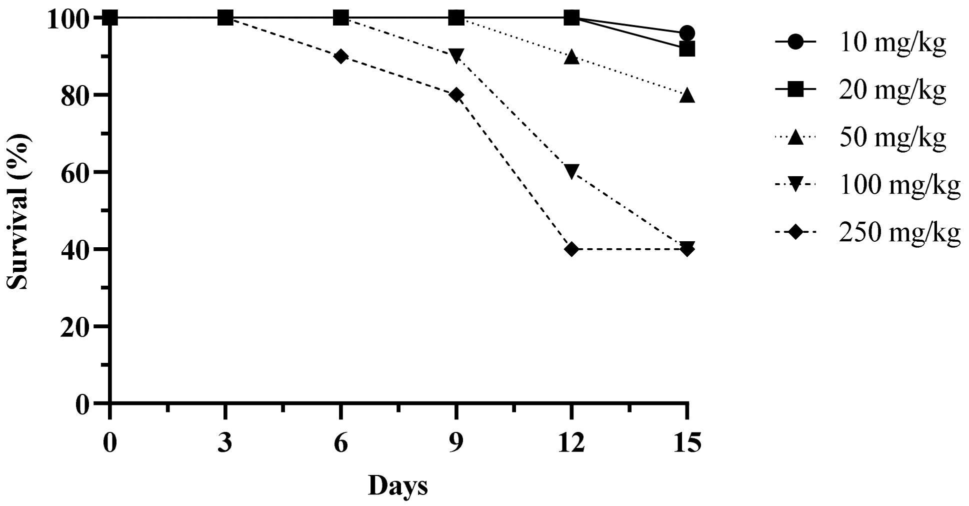

Supplement: Supplementary file 1 [file molecules-28-00290-s001.zip › molecules-2105957-supplementary.pdf]
